# Supplementary material for: Tubulin tyrosination/detyrosination regulate the affinity and sorting of intraflagellar transport trains on axonemal microtubule doublets
Source: Nat Commun. 2025 Jan 26;16:1055. doi: 10.1038/s41467-025-56098-0 (PMC11770126; doi:10.1038/s41467-025-56098-0)
Supplement: Supplementary file 1 — Supplementary Information [file 41467_2025_56098_MOESM1_ESM.pdf]

Tubulin tyrosination/detyrosination regulate the affinity and sorting of intraflagellar transport trains on axonemal microtubule doublets

Aditya Chhatre[1,2,3], Ludek Stepanek[2,4], Adrian Pascal Nievergelt[2], Gonzalo Alvarez Viar[5], Stefan Diez[1,2,3,*], Gaia Pigino[1,2,5,*]

[1] Cluster of Excellence Physics of Life, TUD Dresden University of Technology, 01062, Dresden, Germany

[2] Max Planck Institute of Molecular Cell Biology and Genetics, 01307, Dresden, Germany

[3] B CUBE - Center for Molecular Bioengineering, TUD Dresden University of Technology, 01307 Dresden, Germany

[4] Institute of Molecular Genetics, Czech Academy of Sciences, 14220 Prague, Czech Republic

[5] Human Technopole, 20017 Milan, Italy

* correspondence to: stefan.diez@tu-dresden.de and gaia.pigino@fht.org

Supplementary Figures

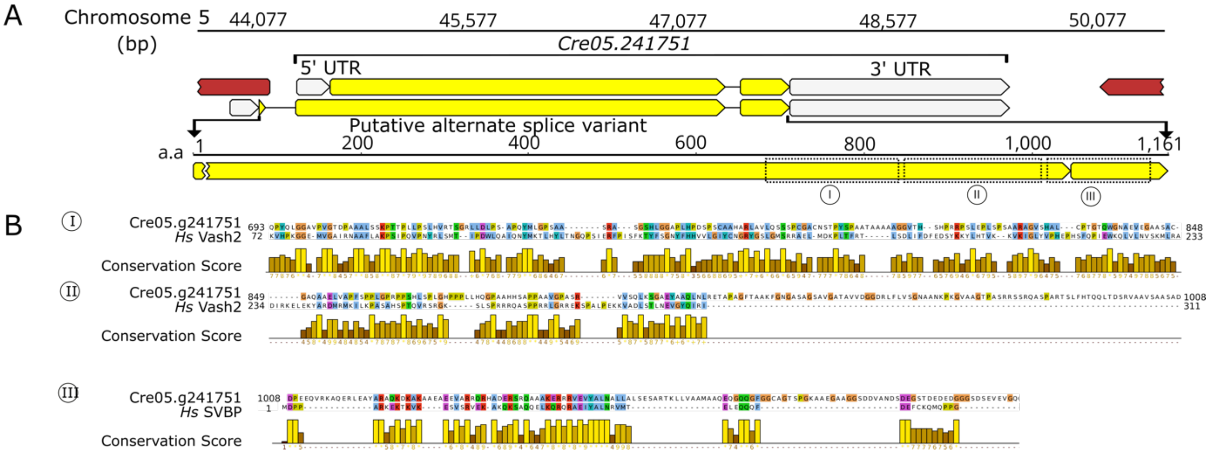

Figure S1. Cre05.g241751 aligns to Human Vash2 and SVBP, related to Figure 1. (**A**) Schematic illustration of *Chlamydomonas reinhardtii* Cre05.g241751 locus on chromosome 5. (**B**) Pairwise sequence alignment of Human Vash2 and SVBP to regions of Cre05.g241751, as marked by arrows.

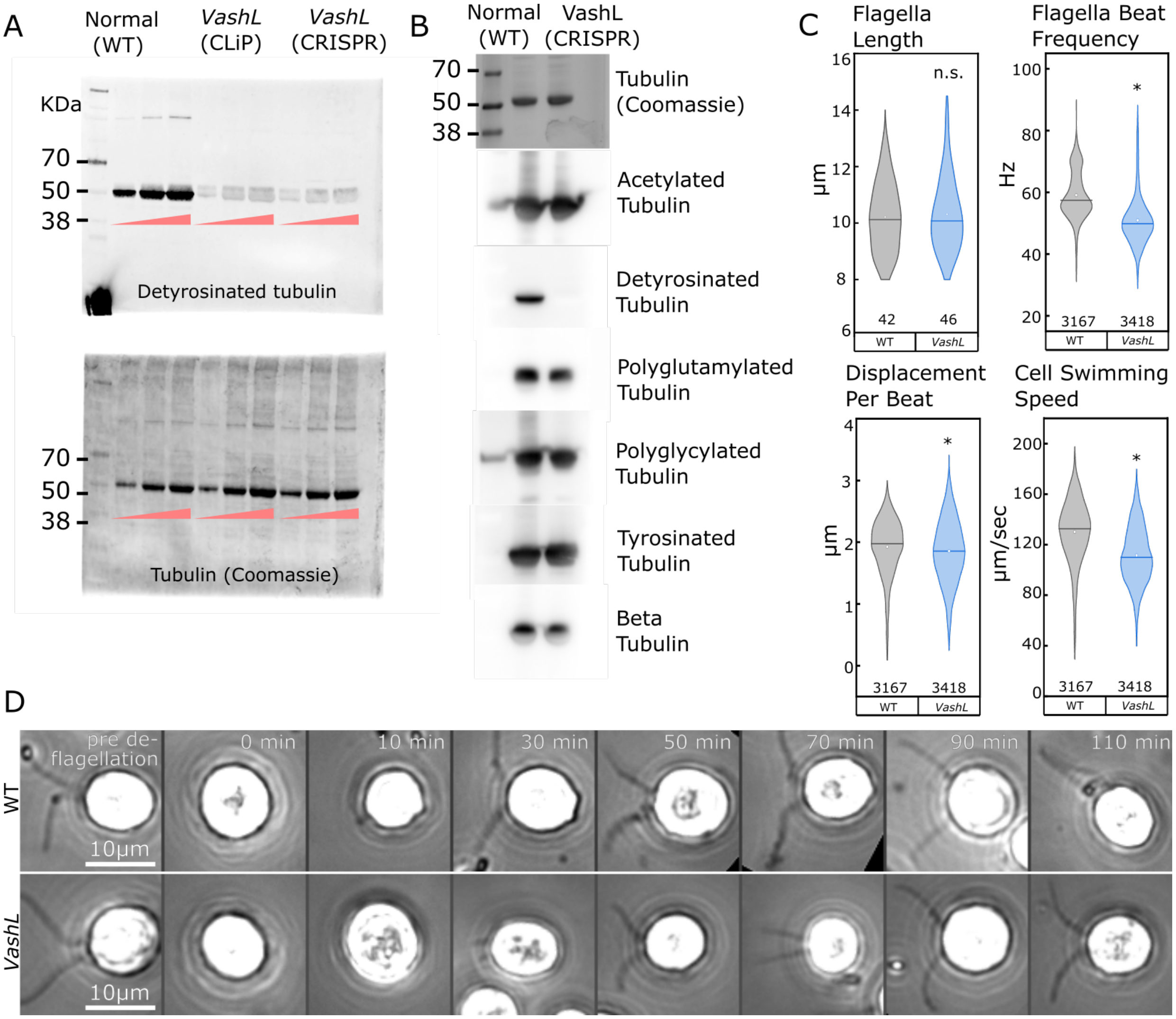

Figure S2. Phenotypic characterization of VashL mutants, related to Figure 1. (**A**) Uncropped western blot for detyrosinated tubulin and Coomassie stain of isolated axoneme of either wild-type/IFT46-mNeonGreen), *VashL* CLiP (LMJ.RY0402.233724), or *VashL* CRISPR mutant. Within each group, axonemes are loaded in 1X, 2X or 3X equivalents. (**B**) Western blot of Wild-type or VashL (CRISPR) axonemes equivalents, probed for various tubulin PTMs as shown (**C**) Violin plots of steady state flagella length, beat frequency, Cell Displacement per beat and swimming velocity, of WT or *VashL* mutant. Total flagella or cells counted from 3 independent cultures. Statistics by Students two tailed t-test. N.s., p-value – not significant. *, p-value <0.05 (**D**) Time course snapshots of ciliary recovery after deciliation, in IFT46-mNeonGreen, with (**Bottom**) or without (**Top**) *VashL* mutation. Quantifications in **Figure 1D**.

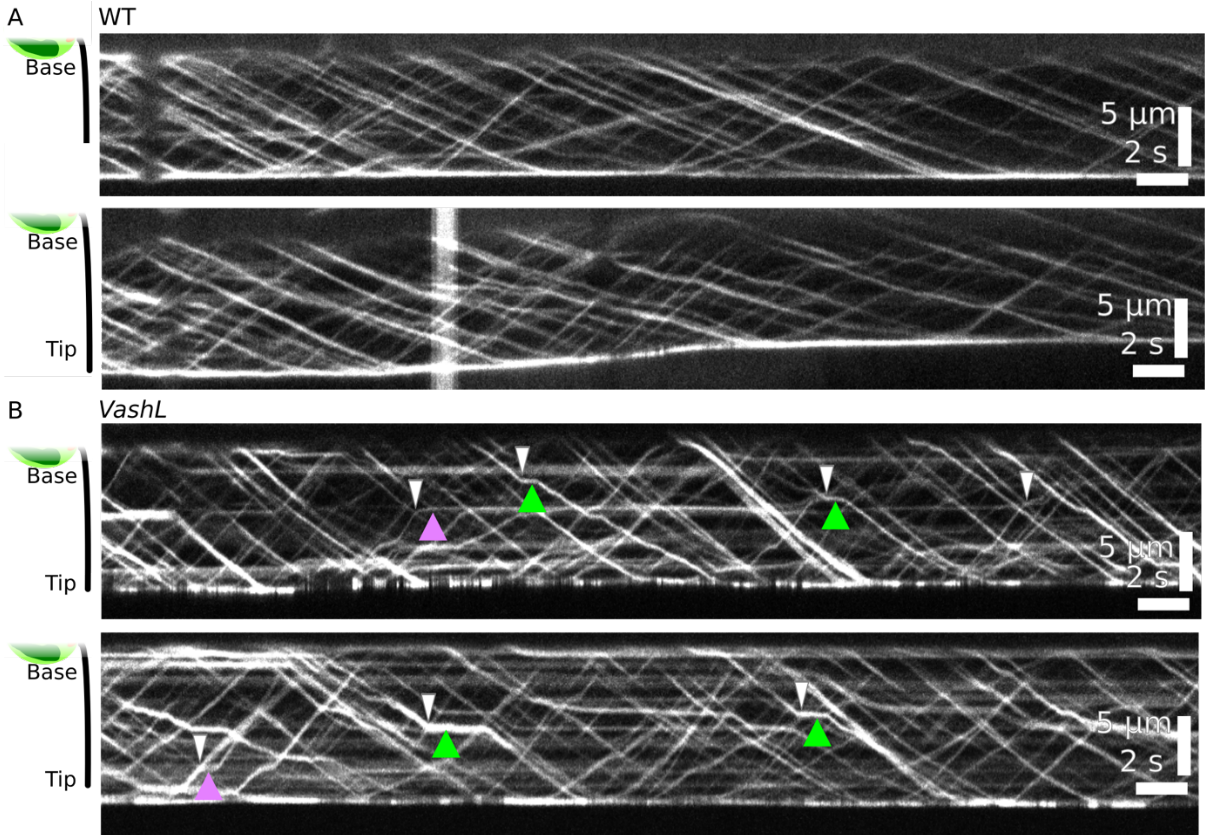

Figure S3. Additional examples of IFT motility in *VashL* cells interrupted by stoppages, related to Figure 1. Kymographs of wild-type (**A**) or *VashL* (**B**) intraflagellar transport (IFT). In *VashL* cells, anterograde or retrograde train stoppages (green or magenta arrowheads) often occur after crossing events (white arrowheads).

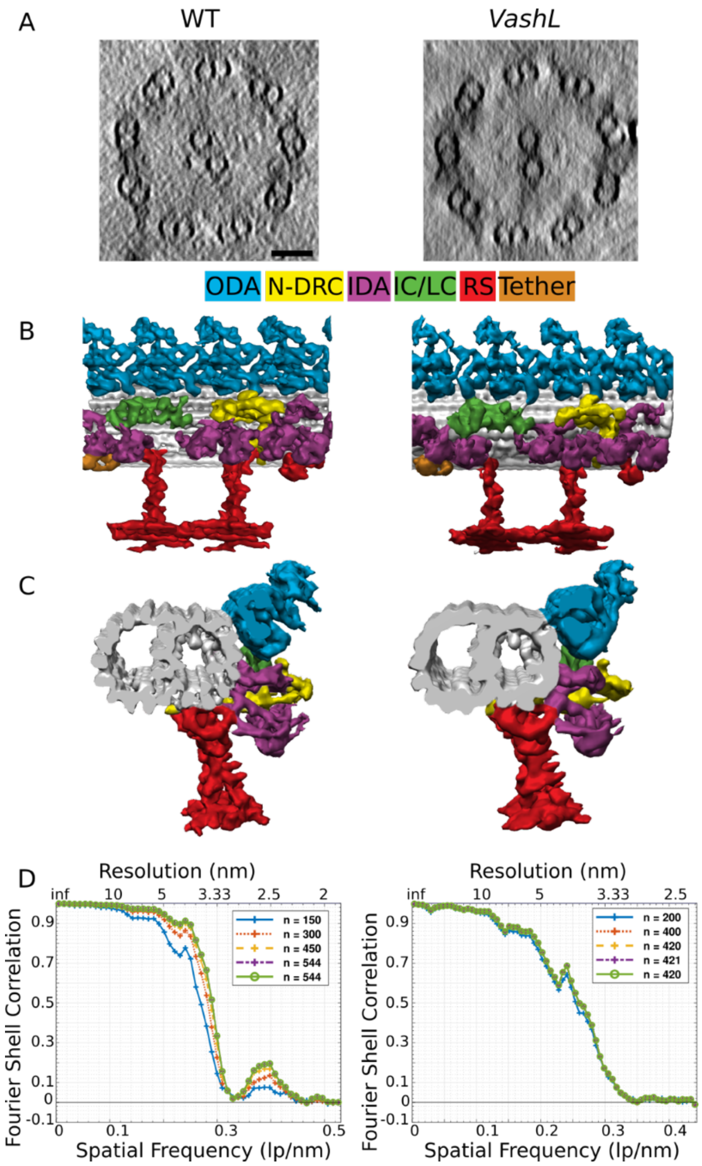

Figure S4: Knock-out of tubulin post-translational modifying enzyme VashL does not alter the macromolecular structure of the Chlamydomonas axoneme.

Characterization of the axonemal structure from different Chlamydomonas strains by cryo-electron tomography. (**A**) Tomographic slice (scale bar 50nm), (**B**) and (**C**) Sub-tomogram averaging electron density model of the 96nm-repeat, (**D**) Fourier shell correlation curves for sub-tomogram averaging structures, of a wild-type (**Left**) or *VashL* (**Right**) Chlamydomonas axoneme. Note that, at macromolecular level, the overall structure of the axonemal 96-nm repeat is unaltered in the *VashL* mutant when compared to wild-type.

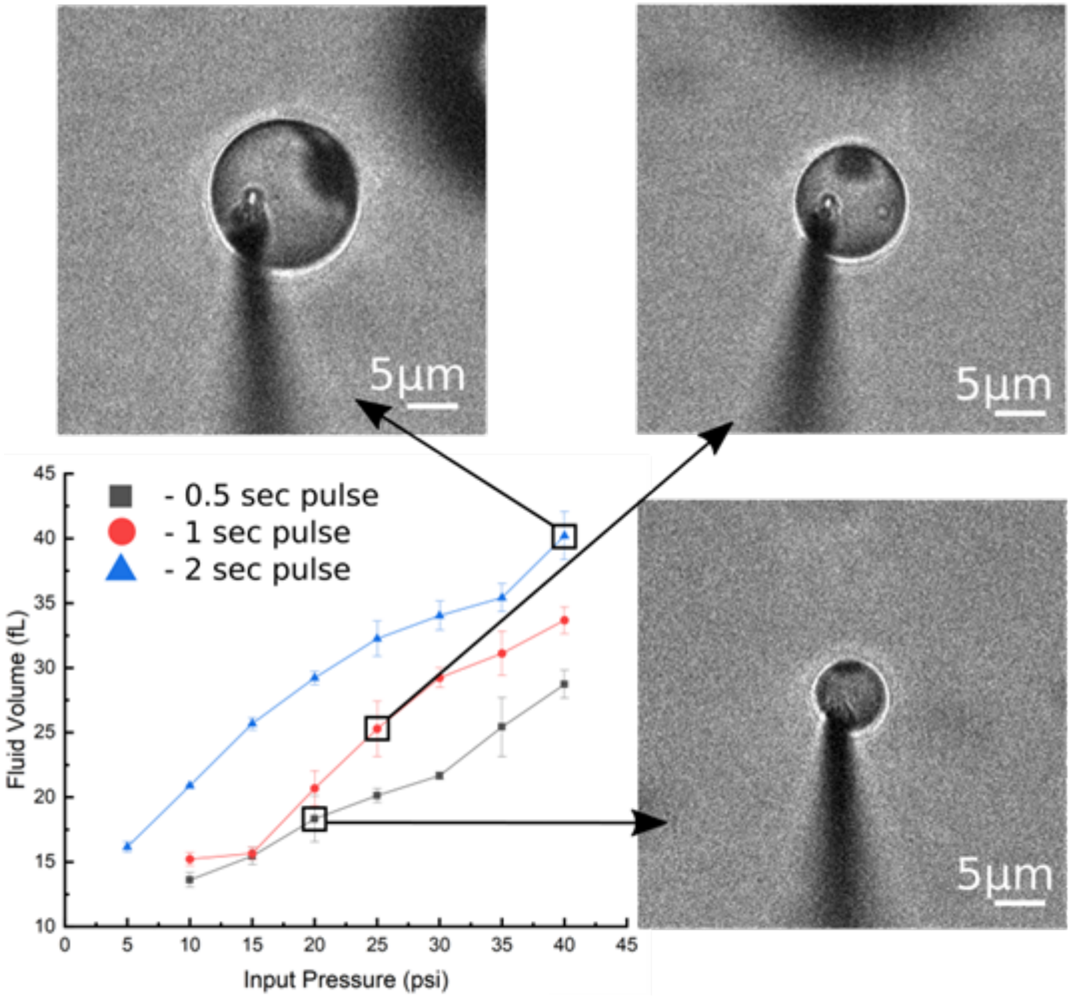

Figure S5. Calibration of microinjector pump. Related to Figure 2 and 3. Volume of ejected fluid (femtoliter) as a function of the input air pressure in injector pump (psi). Volumes estimated from radii of fluid spheres ($1.33*\pi*radius^3$, see Materials and Methods). Stills show representative spheres across the dynamic range of input pressures. Calibrations were performed across N = 3 repeats, average of 3-4 ejections per datapoint.

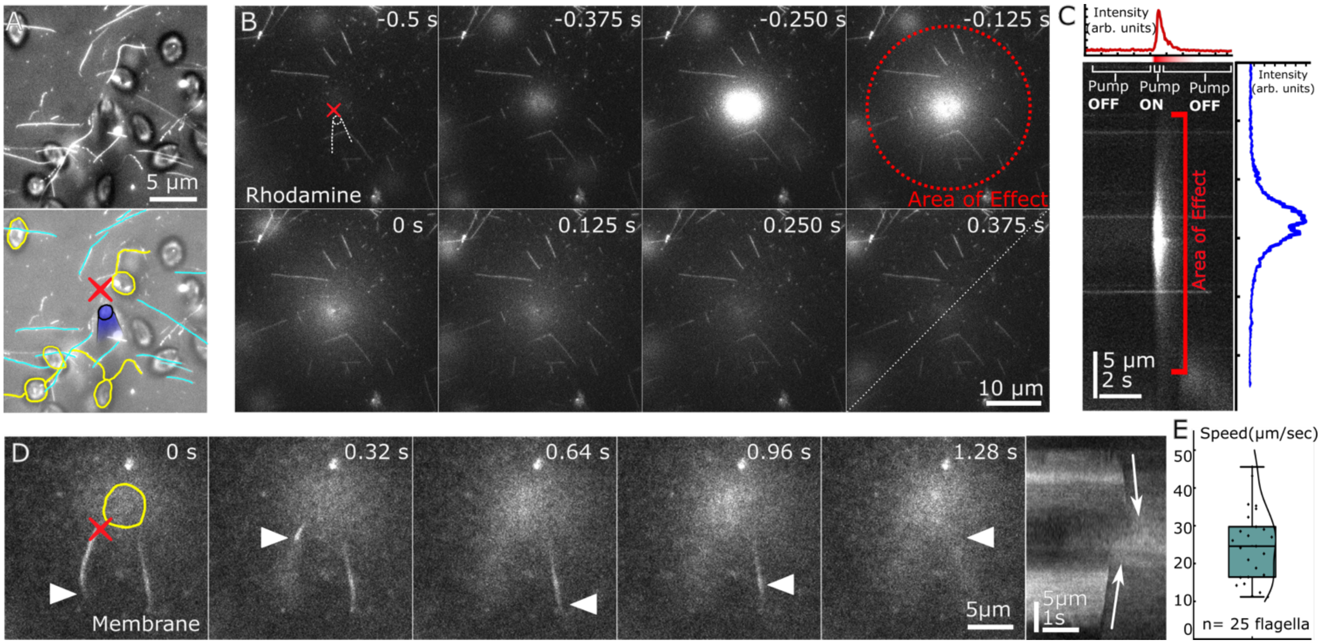

Figure S6. Kinetics of solution ejection and ciliary demembranation, related to Figure 2.

(**A**) Representative transilluminated still of a field of view showing relative positions of the *Chlamydomonas* cells (Yellow), microtubules (Cyan), and overhanging capillary micropipette (blue). Red X marks the spot on the coverslip directly under the needle. (**B**) Time series showing diffusion of liquid (dilute Rhodamine) ejected from capillary pipette over time. The force of ejection does not displace microtubules in the neighbourhood. Relative position and centre of capillary pipette as shown. ‘Area of effect’ is a circle of radius 15 µm with X as the centre. See also **Video S1**. (**C**) Kymograph (white diagonal line in (B)) and two-dimension line intensity plots illustrating kinetics of diffusion of ejected liquid. The concentration of the ejected liquid falls exponentially in space and time from the centre of the needle within the area of effect (red square bracket). (**D**) Montage and kymograph of rapid ciliary demembranation (white arrowheads). Cilia proximal to the centre of the capillary (red X) demembranates first followed by the distal cilia. Cell body traced in yellow for reference. See also **Video S2**. (**E**) Single box plot of sample of the speed of demembranation of cilia as shown in (D). Box bounds represent 25% and 75% quartiles. Line represents median. Box whiskers represent maxima and minima. Each data point indicates individual flagella demembranation event, across 3 biological replicates.

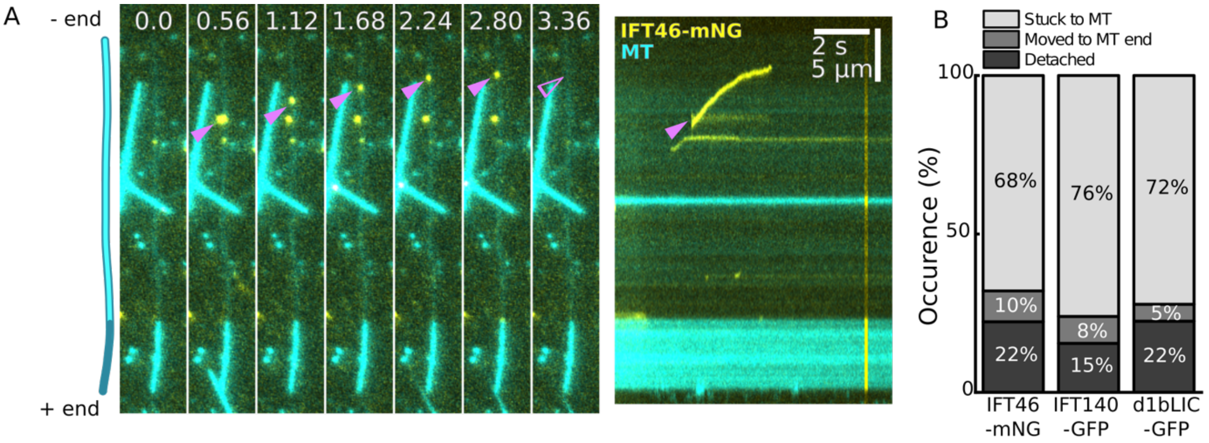

Figure S7. Ex vivo train behaviour on microtubules. Related to Figure 3. (**A**) Montage and example of an ex vivo train that does not halt, but rather detaches from microtubule. (**B**) Percentages of ex vivo train behaviour. Major proportion trains halt on microtubules consistently across multiple samples while a minority detach before halting.

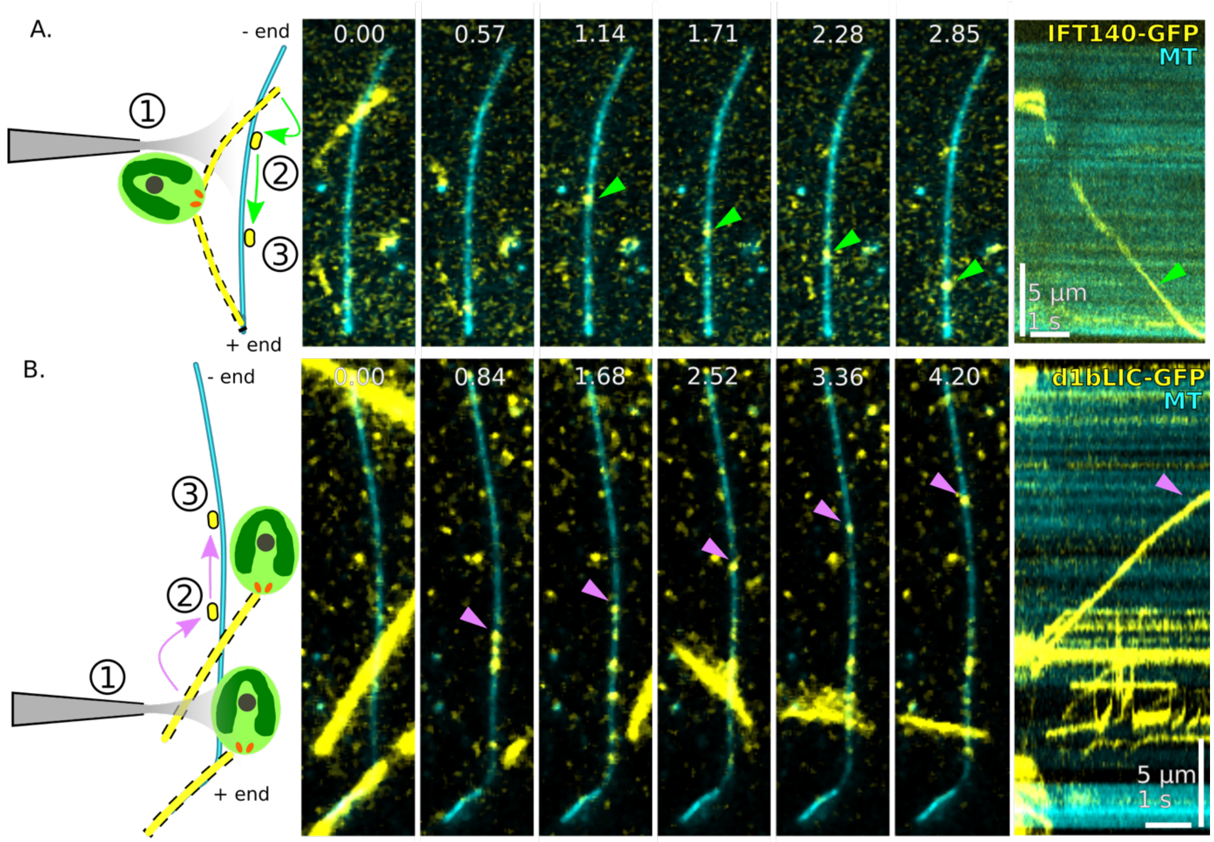

Figure S8. Representative *ex vivo* motility events of IFT trains with labelled IFT-A and IFT-Dynein Complexes. Related to Figure 3. (**A**) **Left:** Schematic of ① detergent shot, ② landing of IFT train and ③ motion on MTs. **Right:** Representative montage and kymograph of reconstitution of IFT train (green arrowhead) from IFT140-sfGFP cells. See also **Video S8**. (**B**) **Left:** Schematic of ① detergent shot, ② landing of IFT train and ③ motion on MTs. **Right:** Representative montage and kymograph of reconstitution of IFT train (magenta arrowhead) from d1bLIC-GFP cells. See also **Video S9**.

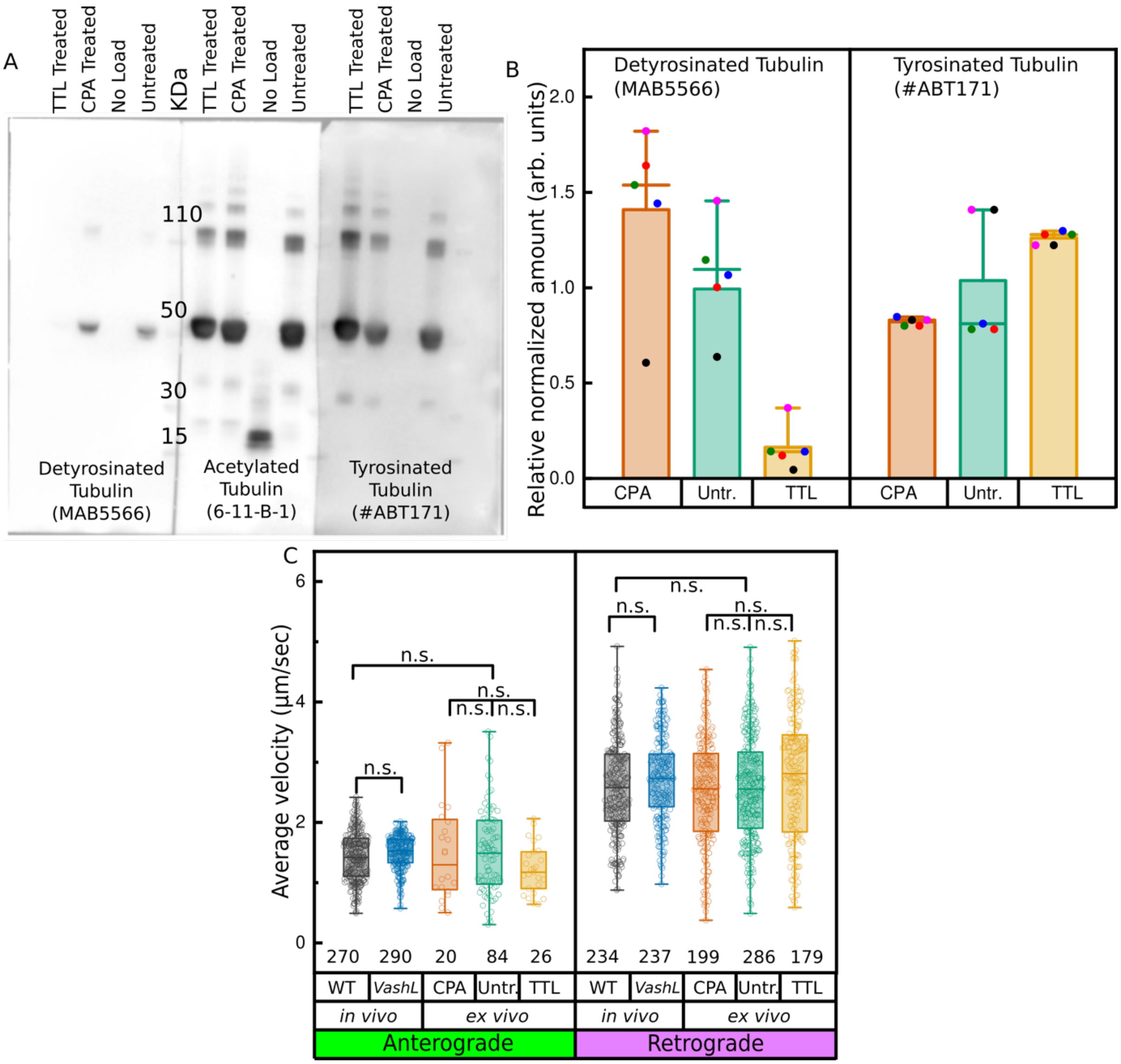

Figure S9. Quantitation of modifications and motility characterization of IFT trains on tyrosinated and detyrosinated microtubules. Related to Figure 5. (**A**) Full uncropped Western blots of identical batches of untreated, carboxypeptidase-A, or tubulin tyrosine ligase treated porcine brain tubulin. Each blot is probed with antibodies specific to either detyrosinated, acetylated or tyrosinated tubulin as mentioned (see Materials and Methods). (**B**) Quantitation and normalization of detyrosinated/tyrosinated tubulin in (A). Samples were normalized w.r.t. acetylated tubulin and further normalized w.r.t. untreated tubulin. Colour scheme in data points of each bar plots represents individual experimental replicates (N=5). (**C**) Box plots for

anterograde and retrograde train velocities, *In vivo* and *Ex vivo.* Total number of trains analyzed as shown. 40-60 flagella and 90-120 demembranations were considered for *in vivo* and *ex vivo* trains respectively from 4-5 independent experimental replicates. *In vivo* estimations exclude stoppage times. Statistics by Student's two tailed t-test. N.s., p-value – not significant.
