# Supplementary material for: Tubulin tyrosination/detyrosination regulate the affinity and sorting of intraflagellar transport trains on axonemal microtubule doublets
Source: Nat Commun. 2025 Jan 26;16:1055. doi: 10.1038/s41467-025-56098-0 (PMC11770126; doi:10.1038/s41467-025-56098-0)
Supplement: Supplementary file 2 — Description of Additional Supplementary Information [file 41467_2025_56098_MOESM2_ESM.docx]

**Description of Additional Supplementary Files**

File Name: Supplementary Movie 1

Description: Uniform diffusion of ejected liquid (dilute Rhodamine) over the area of effect does not displace microtubules in the neighborhood. Related to Figure 2 and Figure S6.

File Name: Supplementary Movie 2

Description: Movie of rapid solubilization of Chlamydomonas ciliary membrane caused by detergent shot (indicated by white circle). Related to Figure 2 and Figure S6.

File Name: Supplementary Movie 3

Description: Representative movie of a experiment showing landing and motility of IFT46-mNeonGreen labelled trains (yellow) on Alexa647-labelled microtubules (cyan; not polarity marked). Cell bodies (cyan; autoflourescence) are shown for clarity. Related to Figure 2 and Figure 3.

File Name: Supplementary Movie 4

Description: Representative TIRFM movie used for determining directionality and velocity of ex vivo IFT46- mNeonGreen labelled trains (yellow), moving on TAMRA-labelled microtubule (cyan; bright (+) end). Related to Figure 3.

File Name: Supplementary Movie 5

Description: Representative TIRFM movie with FMG1b-mNeonGreen labelled (yellow) cells showing no motile events on TAMRA-labelled microtubule (cyan; non polarity marked) upon detergent shot.

File Name: Supplementary Movie 6

Description: Representative TIRFM movie with pf14::RSP3-NeonGreen labelled (yellow) cells showing no motile events on TAMRA-labelled microtubule (cyan; non polarity marked) upon detergent shot.

File Name: Supplementary Movie 7

Description: Representative TIRFM movie with oda6::IC2-mNeonGreen labelled (yellow) cells showing no motile events on TAMRA-labelled microtubule (cyan; non polarity marked) upon detergent shot.

File Name: Supplementary Movie 8

Description: Representative TIRFM movie used for determining directionality and velocity of ex vivo IFT140- sfGFP labelled trains (yellow), moving on TAMRA-labelled microtubule (cyan; bright (+) end). Related to Figure 3 and Figure S8.

File Name: Supplementary Movie 9

Description: Representative TIRFM movie used for determining directionality and velocity of ex vivo d1bLICGFP labelled trains (yellow), moving on TAMRA-labelled microtubule (cyan; bright (+) end). Related to Figure 3 and Figure S8.

File Name: Supplementary Movie 10

Description: Representative TIRFM movie of IFT46-mNeonGreen labelled cells showing continued association and motility of anterograde train (green arrow) on demembranated parent axoneme, as well as detachment of retrograde train (magenta arrow). Related to Figure 4

File Name: Supplementary Movie 11

Description: Representative TIRFM movie of VashL IFT46-mNeonGreen (VashL mutant) cells showing detachment of anterograde train (green arrow) from demembranated parent axoneme, as well as continued association and motility of retrograde train (magenta arrow). Related to Figure 4.
